# Sleep: An Open-Source Python Software for Visualization, Analysis, and Staging of Sleep Data

**DOI:** 10.3389/fninf.2017.00060

**Published:** 2017-09-21

**Authors:** Etienne Combrisson, Raphael Vallat, Jean-Baptiste Eichenlaub, Christian O'Reilly, Tarek Lajnef, Aymeric Guillot, Perrine M. Ruby, Karim Jerbi

**Affiliations:** ^1^Département de Psychologie, Université de Montréal Montreal, QC, Canada; ^2^Inter-University Laboratory of Human Movement Biology, Université Claude Bernard Lyon 1, Université de Lyon Lyon, France; ^3^Lyon Neuroscience Research Center, Brain Dynamics and Cognition team, INSERM UMRS 1028, CNRS UMR 5292, Université Claude Bernard Lyon 1, Université de Lyon Lyon, France; ^4^Department of Neurology, Massachusetts General Hospital, Harvard Medical School Boston, MA, United States; ^5^Blue Brain Project, École Polytechnique Fédérale de Lausanne Geneva, Switzerland; ^6^Center for Advanced Research in Sleep Medicine, Hôpital du Sacré-Coeur de Montréal Montreal, QC, Canada

**Keywords:** polysomnography, electroencephalography, automatic detection, graphoelements, hypnogram, scoring, graphical user interface, opengl

## Abstract

We introduce Sleep, a new Python open-source graphical user interface (GUI) dedicated to visualization, scoring and analyses of sleep data. Among its most prominent features are: (1) Dynamic display of polysomnographic data, spectrogram, hypnogram and topographic maps with several customizable parameters, (2) Implementation of several automatic detection of sleep features such as spindles, K-complexes, slow waves, and rapid eye movements (REM), (3) Implementation of practical signal processing tools such as re-referencing or filtering, and (4) Display of main descriptive statistics including publication-ready tables and figures. The software package supports loading and reading raw EEG data from standard file formats such as European Data Format, in addition to a range of commercial data formats. Most importantly, Sleep is built on top of the VisPy library, which provides GPU-based fast and high-level visualization. As a result, it is capable of efficiently handling and displaying large sleep datasets. Sleep is freely available (http://visbrain.org/sleep) and comes with sample datasets and an extensive documentation. Novel functionalities will continue to be added and open-science community efforts are expected to enhance the capacities of this module.

## Introduction

Polysomnography provides a comprehensive recording of the major physiological changes associated with sleep and is hence the gold standard for modern sleep analysis, both in research and clinical settings. At its simplest, it consists of monitoring at least 2 electroencephalogram (EEG), an electro-oculogram (EOG), and a submental electromyogram (EMG), providing sufficient information to identify sleep stages (sleep scoring) according to standard international established guidelines. A first set of rules were published by Rechtschaffen and Kales (1968) and proposed to divide sleep into 5 stages with distinct electrophysiological properties, named rapid-eye movement (REM) and non-REM (NREM) stages 1, 2, 3, 4. This nomenclature was updated in 2007 by the American Academy of Sleep Medicine (Iber et al., 2007) and sleep stage 3 and 4 have been merged into stage N3. In humans, a normal night of sleep consists of a repetition of four or five cycles in which sleep stages tend to follow each other in a particular order. Sleep staging is generally done visually by inspecting consecutive polysomnographic segments of 30 s. It results in a hypnogram which represents the succession of sleep stages across time. Apart from being time-consuming, visual sleep scoring is subject to both inter and intra-rater variability and is thus far from being optimal. By contrast, automatic sleep scoring has the advantage of being fast, reproducible and with generally good agreement with visual scoring (Berthomier et al., 2007; Lajnef et al., 2015a), yet its usage is far from being widespread and most sleep laboratories still rely on visual scoring, using either commercial softwares or in-house packages. In many cases, these software tools come with their own data and hypnogram file formats, and this heterogeneity can represent a substantial obstacle for sharing of sleep data across laboratories or clinics. Some of the very few existing open sources graphical user interface (GUI) for reading and scoring sleep include Phypno^1^, written in Python, and the MATLAB-based toolboxes sleepSMG^2^ or SpiSOP^3^.

With this in mind, we developed *Sleep*, an intuitive and efficient open-source GUI dedicated to the visualization of polysomnographic recordings and scoring of sleep stages. *Sleep* supports a range of data file formats and provides several scoring aid including the detection of essential features of NREM and REM sleep such as spindles, K-complexes, slow waves, and REM. *Sleep* was written in Python, an easy-to-learn high-level programming language widely used in the scientific community. We developed *Sleep* on top of VisPy^4^ (Campagnola et al., 2015), a Python scientific library based on OpenGL which offloads graphics rendering to the graphics processing unit (GPU) in order to provide fast and high-quality visualization, even under heavy loads as is the case with large dataset. *Sleep* therefore benefits from the high performances provided by VisPy alongside Python's inherent qualities such as its portability and ease of use.

## Methods

Scientific visualization often consists of finding the best possible way to explore the data and to illustrate results in an intuitive and straightforward manner. The huge variety of neuroscientific data types and acquisition modalities naturally requires a wide range of specific visualization tools. Ideally, software packages needed for the various applications should be free and capable of handling several types of brain data recordings. In this context, we are currently developing a Python package we called Visbrain^5^ distributed under a BSD license, which provides and centralizes a number of useful brain data visualization utilities. Given the lack of software solutions that wrap together a portable and user-friendly interface for polysomnographic data visualization and edition, we set out to develop an open-source module (included within the Visbrain package) and named Sleep.

### The choice of python and the project vision

The choice of the programming language naturally turned to Python as this high-level and open-source language benefits from many libraries, an extensive documentation and a dynamic community. From data analysis to the production of high-definition paper figures, Python offers all the tools needed by scientists, with the comfort of a clean and easy to read syntax. *Sleep* is a pure Python software built on top of NumPy, VisPy, PyQt4^6^ and uses a limited number of functions from SciPy and Matplotlib. Thanks to the Python portability, the software can be installed and used on any platform. One of the initial objectives of the project was to provide a user-friendly and intuitive interface capable of loading and displaying large sleep dataset. To this end, we paid a particular attention to avoid deep data copy and display only what is necessary. Therefore, even very large recordings with a consequent number of channels can be handled by *Sleep* on any modern laptop with snappy GUI response. From a programming perspective, we did our best to provide a clean, commented and high-quality code, with a NumPy style documentation and using static analysis tool, as recommended by PEP 8. *Sleep* is hosted on GitHub and we encourage Python programmers and sleep scientists to collaborate in order to collectively improve this software by extending its functionalities and data compatibilities.

### Hardware accelerated graphics

In addition to ergonomic considerations and providing a portable interface, a further important goal was to use a plotting library which would allow our *Sleep* module to support and process large sleep data. Using Matplotlib was an option we considered, but although it is particularly convenient to produce publication quality figures, it is not the best option when it comes to plotting and interacting in real-time. In contrast, VisPy is a scientific visualization library based on NumPy and OpenGL and was primarily designed to provide both high performances with real-time interactions and publication quality figures. VisPy provides a bridge between the intuitive Python syntax and modern shader-based OpenGL pipeline allowing the graphical rendering cost to be offloaded to the GPU. This package has been well-designed and is built on four levels, from a Matplotlib oriented one to the lowest-level (closer to OpenGL) which makes it more flexible and efficient at the cost of a potentially slower learning curve. Because all *Sleep* graphical elements are primitive 2D objects (line, points, and images) it was not a necessity to go down to the lowest level of VisPy (vispy.gloo). Indeed, all required objects were already implemented into the Visual library. Hence, any modern computer equipped with a GPU should see the benefits of the hardware accelerated graphics implemented in *Sleep*.

### Portable GUI through python

Currently, among the major cross-platform GUI toolkits that interface with Python, wxWidgets^7^ (wxPython), Tcl/Tk^8^ (TkInter), and Qt^9^ (PyQt/PySide) are probably the most known and used. We chose PyQt which is a python binding for the C++ Qt toolkit, and we used Qt Designer to design the GUI.

Taken together, VisPy provides high-performance rendering graphics that are well-integrated in a portable, modular and responsive Qt GUI using Python PyQt package. The use of this library is therefore one of the major strengths of this open-source module, and is particularly important when it comes to handling large multi-dimensional brain data, such as full-night sleep EEG recordings.

### Automatic events detection

**Figure 1:**
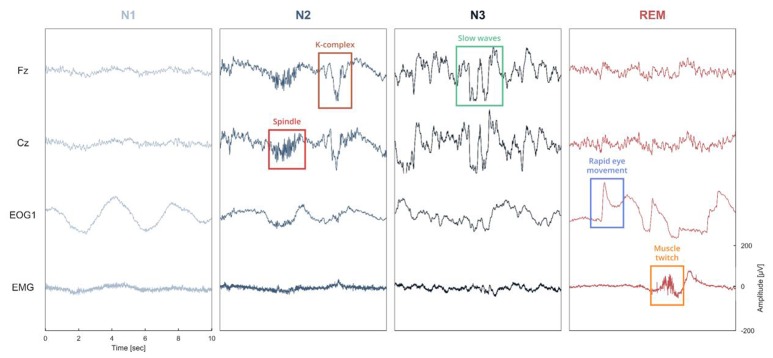
Illustration of the different sleep features observed in a polysomnographic recording of one individual. To see examples of automatic detection actually performed by our software, see Figure 4. Spindles and K-complexes are landmarks of N2 sleep. Slow waves are present during N3 sleep (sometimes referred to as slow wave sleep). Rapid eye movements, observed in the EOG channel, and muscle twitches, observed on the EMG channel, are two essential features of rapid eye movement (REM) sleep.

One of the main objectives of *Sleep* was to provide a complete and easy-to-use interface for analyzing and staging sleep data. To this purpose, we implemented several algorithms for the automatic detection of sleep features, and embedded them within the software (“Detection” panels). This includes detection of spindles, K-complexes, slow waves, rapid-eye movements, muscle twitches, and signal peaks. With the exception of the latter, all these features are often used as landmarks of specific sleep stages and can be very helpful to guide experts in their identification of specific sleep stages within a period of sleep, i.e., sleep scoring or sleep staging (see Figure 1). The main characteristics of each of these features are summarized below.

**Figure 2:**
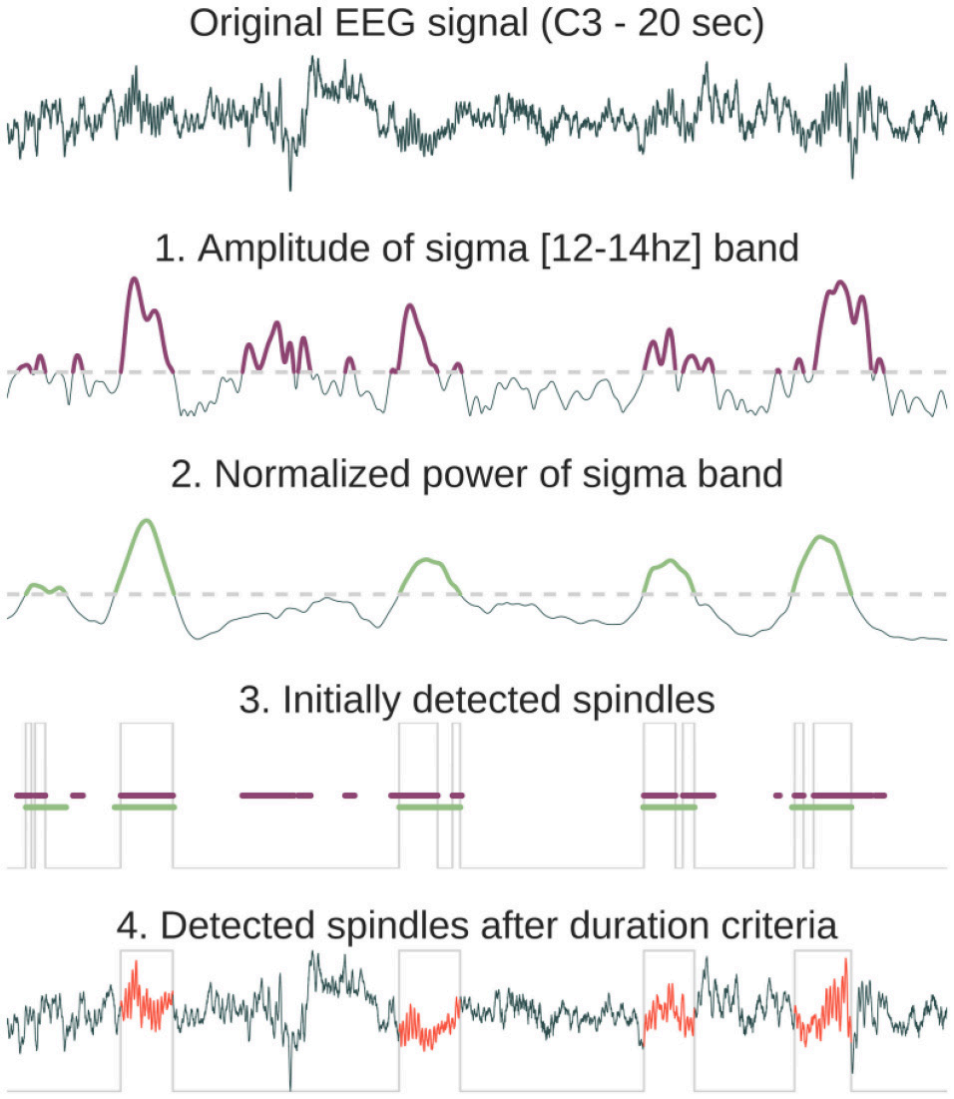
Method description for the automatic sleep spindles detection. First, the original signal is convoluted with a Morlet wavelet centered in the spindles frequency band [12–14 Hz]. From the resulting complex decomposition, we only keep the amplitude and find time indices where the amplitude exceeds the threshold (purple in 1). Then, we compute the normalized power in the sigma band and detect again time indices where the power exceeds a threshold (green in 2). The normalized sigma power is obtained by first computing absolute power in four frequency bands (delta = [0.5–4 Hz], theta = [4–8 Hz], alpha = [8–12 Hz], sigma = [12–16 Hz]) and then dividing each of them by the sum of these powers. As a result, for each time point the sum of powers in the four frequency bands equals 1. The time location of the initial detected spindles (gray line in 3) is the result of the intersection of exceeding both the amplitude index (purple line) and the power index (green line). Finally, time gaps are filled only for neighboring detected events (<500 ms) and a final duration criteria is applied in order to suppress events with a duration inferior to 500 ms or superior to 2,000 ms (these thresholds can be set within *Sleep* interface, 4).

- Sleep spindles refer to burst of 12–14 Hz waves predominant over central scalp electrodes and lasting between 0.5 and 2 s (Rechtschaffen and Kales, 1968). These bursts of oscillatory activity have been known as a defining characteristics of N2 sleep (although there is an increasing number of studies that analyze spindles in N3 stages). Several automatic spindle detection algorithms have been developed in recent years (reviews in Devuyst et al., 2011; Warby et al., 2014). The algorithm implemented in *Sleep* is based on a wavelet transform followed by amplitude threshold and duration criteria. The default algorithm parameters (duration, frequency, and power threshold) were chosen according to previously published detection methods (Devuyst et al., 2011). The consecutive steps of the spindles automatic detection algorithm implemented in *Sleep* are detailed in Figure 2.
- K-complexes are defined as sharp negative waves followed by a positive component, prominent over frontal scalp electrodes and lasting more than 0.5 s. Along with spindles, they constitute one landmark of N2 sleep. Briefly, the algorithm implemented in *Sleep* comprises the following steps: (1) bandpass filtering of the signal in the delta frequency band (2) amplitude thresholding of the Teager-Keaser Energy Operator (Erdamar et al., 2012; Parekh et al., 2015) of the filtered signal (3) computation of the probability of detecting true K-complexes based on morphological criteria (duration and amplitude) and the presence of spindles in the vicinity.
- Slow-waves (or delta waves) are high-amplitude (>75 μV) and low-frequency (<3 Hz) oscillations that are present during the deepest NREM sleep stage, i.e., N3 sleep. According to the standard international guidelines, N3 sleep is defined by the presence of 20% or more slow waves in a given epoch. As period of N3 sleep are marked by a high delta power and low power in the other frequency bands (theta, alpha, beta), the algorithm implemented in *Sleep* is based on a thresholding of the delta relative band power.
- As its name suggests, REM sleep is characterized by rapid eye movements easily observable on the EOG channels. They consist of conjugate, irregular and sharply peaked eye movements, similar to some extent to those exhibited during wakefulness. The algorithm implemented for the detection of REMs is detailed elsewhere (Vallat et al., 2017).
- Another fundamental aspect of REM sleep is its muscle atonia, as revealed by a low EMG activity. However, some transient muscle activity or muscle twitchings (MTs) can also be observed. These short irregular bursts of EMG activity are superimposed on the background of low EMG activity. The automatic detection of MTs is based on a thresholding of the Morlet's complex decomposition of the EMG signal followed by morphological criteria (duration and amplitude).
- Finally, *Sleep* implements a signal peak detection algorithm that is useful for example to calculate the heart rate, provided that an ECG channel is present. The algorithm implemented in *Sleep* searches for the highest point around which there are points lower by a certain duration on both sides^10^.

Altogether, the set of detectors implemented in our software offers a valuable help for scoring sleep stages through the identification of the main features of each sleep stages. Detections can also be used for a more in-depth analysis of the sleep microstructure (e.g., Vallat et al., 2017). Comparisons of performances between our detections and visual scoring are reported for K-complexes and spindles in the Results section.

### Signal processing tools

In addition to the automatic detections presented above, *Sleep* also provides a wide range of basic and advanced signal processing tools such as signal demeaning, detrending, and a filtering. The latter can be done either with Butterworth or Bessel filters and four filter designs are currently available: lowpass, highpass, bandpass, or bandstop. Importantly, further information can be extracted from the Morlet's wavelet complex decomposition (Tallon-Baudry et al., 1996) such as time-resolved and band-specific amplitude, power or phase. Critically, each one of these signal processing tools are reversible and can therefore be activated and deactivated without altering the original data and without any memory-intensive data copy. Finally, loaded data can be re-referenced directly from the interface by either re-referencing to a selected single channel or common-average (frequently used for scalp EEG datasets) or by using bipolarization, which consists of subtracting neural activity from consecutives sites (classically used in intracranial EEG, see Jerbi et al., 2009).

### Documentation and examples

*Sleep* comes with a detailed step-by-step documentation, built with Sphinx^11^ and hosted on GitHub^12^. This documentation include a description of the graphical components and the main functionalities of the software. A PDF version of the documentation can also be downloaded from the “Help” contextual menu of the software. We also provide anonymized and free-to-use sample datasets, including the corresponding loading scripts. This will help users test the *Sleep* module and get familiar with its functionalities before trying it on their own data. Finally, we also implemented an interactive documentation using the tooltips provided by PyQt to describe each element of the interface.

## Results

In the following we overview the current GUI and software functionalities of Sleep and provide details on hypnogram editing and event detection validation results.

### Graphical user interface

**Figure 3:**
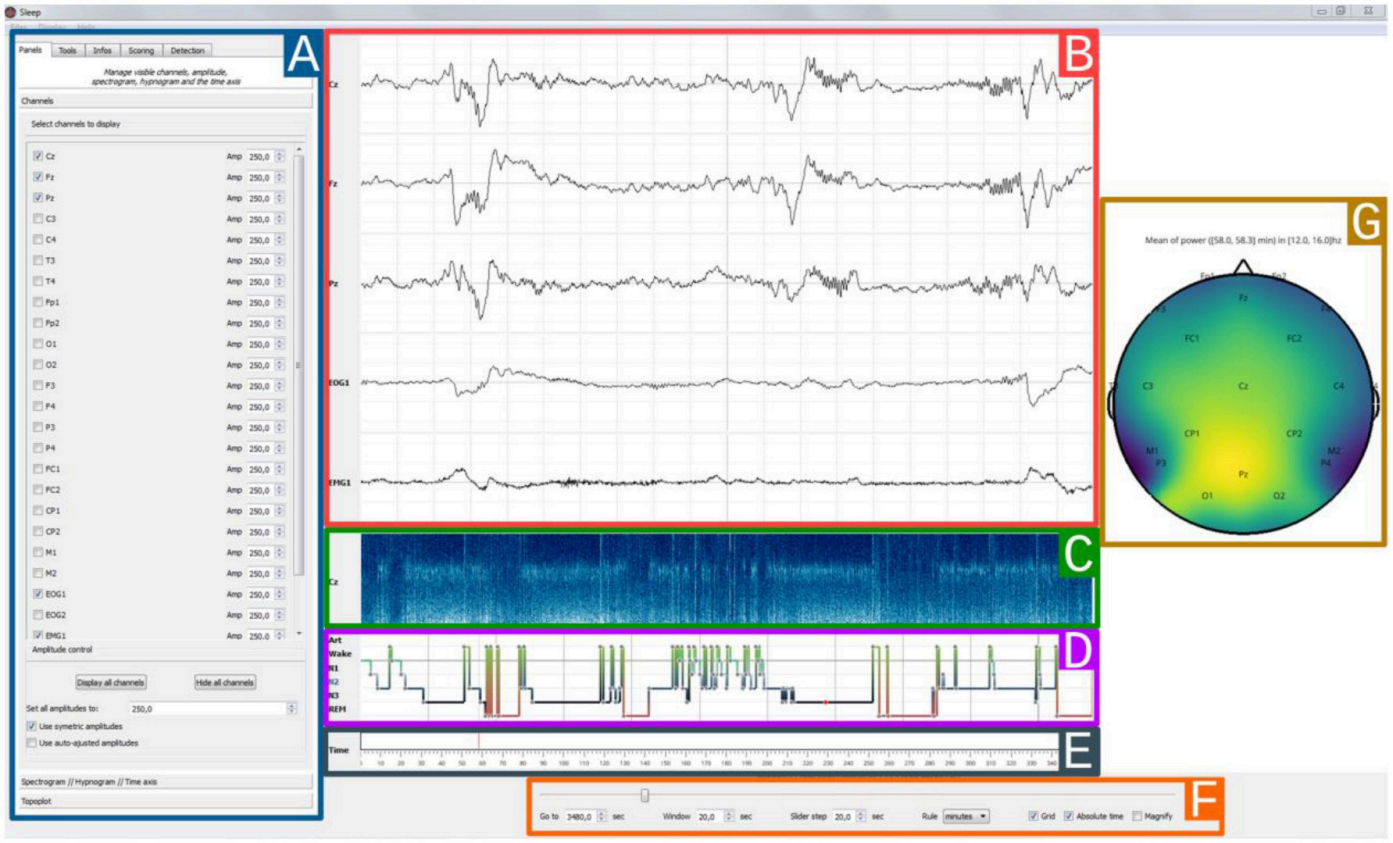
*Sleep* main interface. Each element of the graphical user interface can either be displayed or hidden, **(A)** Settings panel containing all *Sleep* controls and parameters. The current displayed tab can be used to toggle channel visibility and to adjust individual amplitudes, **(B)** 30 s time window of electrophysiological data. Here, only 5 channels are currently displayed (Cz, Fz, Pz, EOG1, EMG1), **(C)** The spectrogram displays the time-frequency representation of a specific channel for the entire recording, and can be useful to identify global changes in the spectral properties of the signal often associated with changes in sleep stages. Any channel can be picked and further time-frequency controls are available in the settings panel, **(D)** Hypnogram with one specific color per stage. The stage order can be changed from the default *Artefact, Wake, REM, N1, N2, N3*, **(E)** Time axis with visual indicator, **(F)** Navigation bar with time settings: window length and step size, unit (seconds/minutes/hours), **(G)** Topographic data representation.

The *Sleep* GUI is currently subdivided into six distinct components (Figure 3): (*1*) settings panel, (*2*) navigation bar, (*3*) hypnogram, (*4*) electrophysiological time series, (*5*) spectrogram canvas, (*6*) topographic map. As the user interface is built up in a modular way, each of these components can be hidden or displayed, depending on whether the user prefers a light or fully-featured interface. Using the contextual menu, users can save and subsequently load the current display properties in order to easily retrieve and continue working on a previous session.

#### Settings panel and navigation bar

All controls and properties are grouped in a settings panel. This panel is subdivided into five thematic tabs:

- Panels: manage the visibility and properties of each plotted canvas.
- Tools: bundle of signal processing tools.
- Infos: basic informations of the current recording (name, sampling rate) and sleep statistics computed using the hypnogram (sleep stage duration, latency, etc.). Note that the statistics can be exported in ^*^.csv or ^*^.txt file and are automatically updated when the hypnogram is edited.
- Scoring: scoring table that can be used to inspect and edit where each stage starts and end. This panel represents one of the three methods available within the software to edit the hypnogram (see hypnogram edition section) and may be useful for example to score long periods of continuous and homogenous sleep by just providing the starting and ending times.
- Detection: perform and manage the automatic detection of several sleep features.
- Annotations: add notes or comments to specific time points within the recordings. Annotations can be saved and loaded using the File contextual menu or can be passed as an input parameter. Each annotation is then referenced in a table comprising the start and end time (in seconds) and the corresponding text. Selecting a row in the table centers the main window around it. This latter feature enables a quick access to annotated events for a faster navigation. Annotated events are also identified in the time axis as a green triangle.

**Figure 4:**
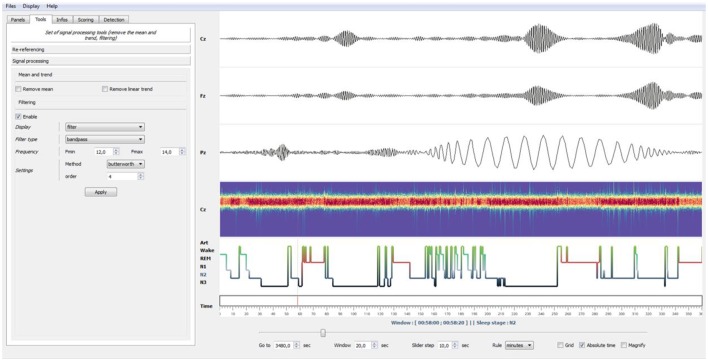
Example of bandpass filtering. Using the Tools panel (**left**), the EEG signals have been bandpass-filtered in the spindles frequency band (12–14 Hz, Butterworth filter). Using the “Enable” checkbox of the panel, this filtering operation can be disabled at any moment to retrieve the original EEG signals. Finally, by left-clicking on a specific time point in a channel or selecting the Magnify tools (**bottom**), users can enlarge events. This was used in this example to enlarge a sleep spindle observed on channel Pz.

In addition to this setting panel, *Sleep* provides a navigation bar that can be used to set several temporal properties, such as the length of the current time window, time step between each window, time units and the use, if provided, of the absolute time of the current recording. This navigation bar also includes a *grid toggle* button that can either hide or display the grid, as well as a *magnify* option to enlarge short events (see Figure 4).

#### Electrophysiological time series

*Sleep* offers a dynamic control of the displayed polysomnographic time series and most of the settings are in the “Panels” tab. Indeed, each channel can be added or removed from the list of the currently displayed canvas. By default, *Sleep* displays the time series by frames of 30 s, which is a standard duration for stage scoring (Iber et al., 2007), but this value can be changed directly from the navigation bar. Furthermore, the amplitude of each channel can either be set independently, using a same range across all channels, or automatically adjusted according to the minimum/maximum of the currently displayed signals.

#### Time-frequency representation

The visibility and amplitude of each channel can be controlled from the GUI (see Figure 3). The same applies for the spectrogram, which corresponds to a time-frequency representation of the entire recording performed on one channel. Among the definable parameters of the spectrogram are the channel on which it is computed, lower and upper limit frequencies, length and overlap of the fast Fourier transform and colormap properties. Finally, a topographic map based on the Source Connectivity Toolbox (SCoT) and the Magnetoencephalography and Electroencephalography (MNE) toolbox implementations (Gramfort et al., 2013; Billinger et al., 2014) can also be embedded inside the GUI for full data inspection. The topological plot depicts the mean values computed from the time window currently displayed. This channel-space 2D topographical functionality provides a convenient and versatile tool to visualize various data types, including the raw data, the amplitude or power in specific frequency bands.

#### Shortcuts

Navigation and operations inside a software can be sometimes repetitive. For that reason, *Sleep* comes with numerous native shortcuts to facilitate the visualization and stage scoring. For a complete list we refer the reader to the “Shortcuts” paragraph of the documentation^13^.

### Supported electrophysiological and hypnogram data formats

*Sleep* natively supports several standard electrophysiological file formats, including European Data Format (EDF ^*^.edf), Micromed (^*^.trc), Brain Vision (^*^.eeg), and Elan (^*^.eeg). In addition, it is possible to load directly NumPy array or Matlab file using the command-line parameters.

The hypnogram of the corresponding dataset can also be loaded and then edited directly from the GUI. Accepted hypnogram file formats are ^*^.txt, ^*^.csv, or ^*^.hyp. There is a great heterogeneity among sleep laboratories with respect to hypnogram format. This represents an obvious barrier for data sharing. To overcome this problem, *Sleep* allows the user to specify the hypnogram format in a separate text file. This file should contain the names and integer values assigned to each sleep stage in the hypnogram file, as well as the number of values per second. During loading, the hypnogram file will be converted to *Sleep* native hypnogram format described in the documentation^14^. An example description file can be found in the documentation^14^.

### Editing the hypnogram

The hypnogram can be edited either from scratch or from an existing hypnogram file. There are three methods to edit the hypnogram using *Sleep* GUI:

- Using intuitive keyboard shortcuts. When a new stage is entered, the next window is shown.
- Using a table where each stage can be specified by it starting and ending time point.
- Using a drag and drop operation directly on the hypnogram canvas.

**Figure 5:**
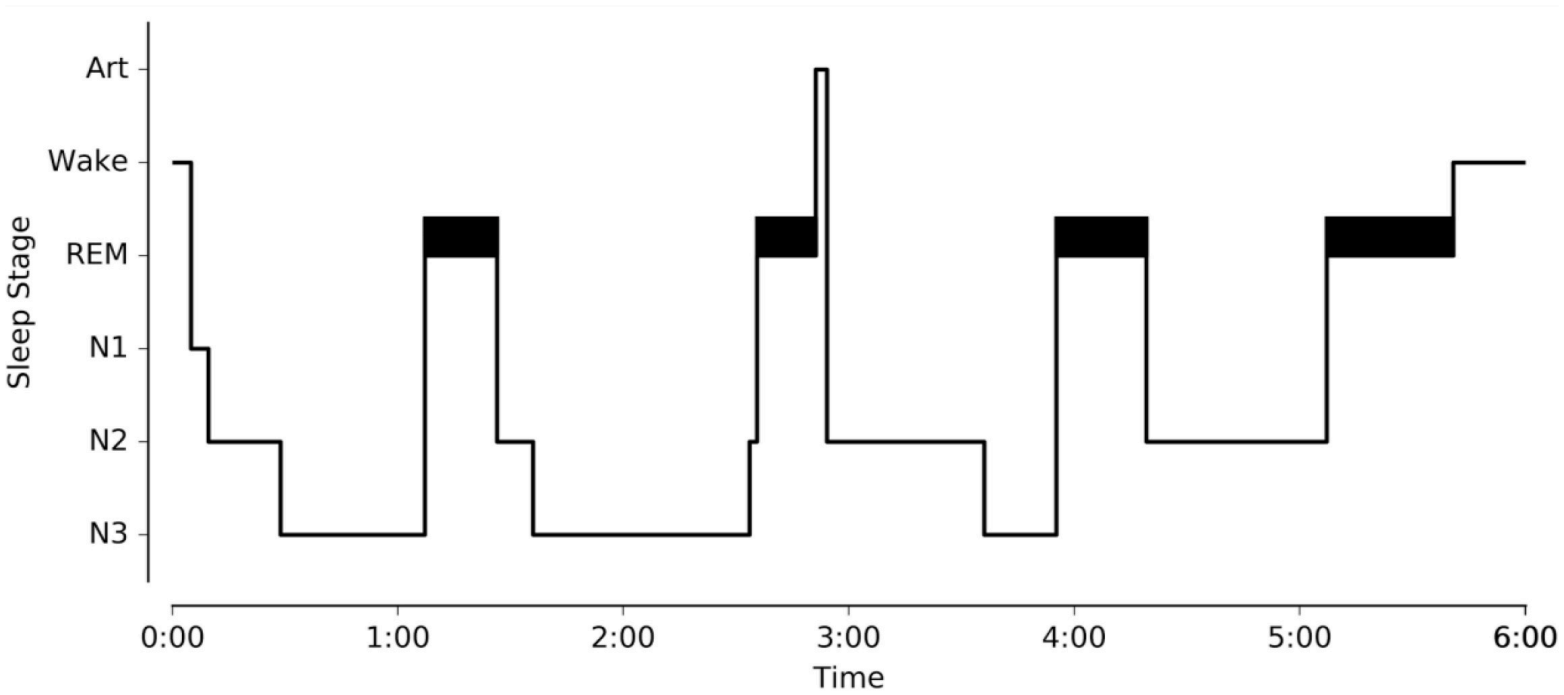
Example of publication-ready hypnogram figure exported using *Sleep* GUI.

At any moment, the user can export the hypnogram or save it as a black and white (or color) publication-ready figure using the contextual menu (Figure 5).

### GUI integration and validation of automatic events detection

**Figure 6:**
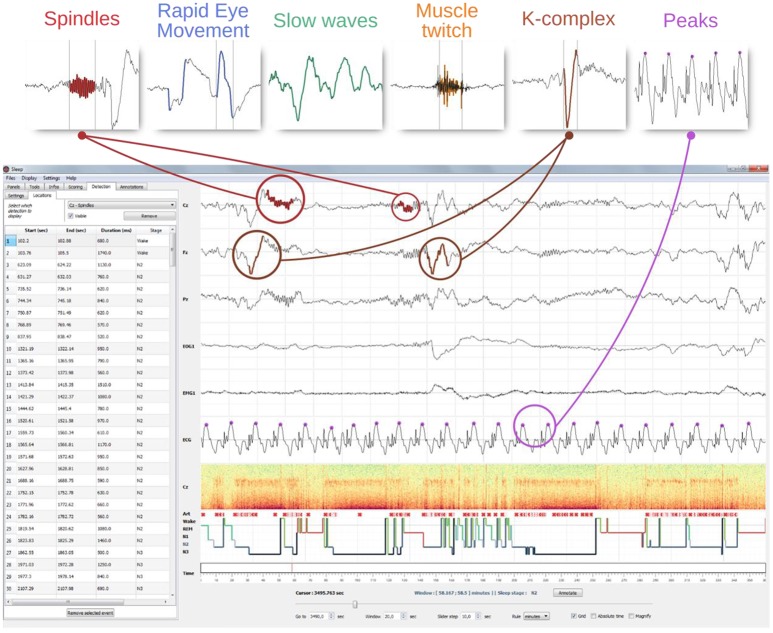
GUI integration of the automatic event detection. The top row illustrate examples of typical graphoelements detected with *Sleep* including spindles, K-complexes, rapid eye movements, slow-waves, muscle twitches, and peaks. The window below illustrate how detections of such events are visually integrated into the interface. First, each detected event are highlighted into the channel time-series. Then, all the detected events are displayed on top of the hypnogram (identified using different symbols and colors per detection type) and reported into a table embedded into the settings panel. A mouse click on a line centers the corresponding event on the screen. This table can be exported into a ^*^.csv or a ^*^.txt file.

The automatic events detection can be performed on any selected or visible channel. When the detection is completed, detected events are depicted directly on the selected channel using a specific color-code for each feature. In addition, the starting point, duration and stage of occurrence of each one of the detected events are reported in the “Location table.” Users can then easily navigate between the detected events by clicking on a row, which automatically sets the time so that the event is centered on the screen. Furthermore, this table can be exported to a ^*^.csv or ^*^.txt file. Users can perform an unlimited number of detections in a row on a single channel and then switch from one to another using the “Location” panel. Last but not least, the location of each detected event is reported on the hypnogram using specific visual cues for each detection types. Integration of the detection inside the GUI is shown in Figure 6.

To test how these detections performed on real datasets, we measured performances of the spindle and K-complex detection methods using visually-annotated EEG segments of N2 sleep collected from full-night polysomnographic recordings of 14 participants (Eichenlaub et al., 2012, 2014; Ruby P. et al., 2013; Ruby P. M. et al., 2013). Spindles and K-complexes were visually scored by an expert (JBE) as part of a previous work that focused specifically on the detection of these sleep features using machine-learning (Lajnef et al., 2015a).

To perform the detection methods using *Sleep* algorithm, all N2-sleep EEG segments were concatenated into a single file of 210 min with a single channel (C3) and with a sampling rate of 100 Hz (native downsampling frequency of *Sleep*). Then, to evaluate the performances of our detection, we used two standards metrics: the sensitivity *(1)*, which measures the proportion of correctly identified detected events and the False Detection Rate (FDR) *(2)* which assess the proportion of incorrectly detected events.

(1) $$Sensitivity\;=\;\frac{True\;Positive}{True\;Positive\;+\;False\;Negative}$$

(2) $$False\;Detection\;Rate\;=\;\frac{False\;Positive}{False\;Positive\;+\;True\;Positive}$$

where True Positive refers to the events scored by the expert and correctly detected by our methods, False Negative refers to the events scored by the expert but not detected by our method and False Positive refers to the events detected by our methods but not scored by the expert.

**Figure 7:**
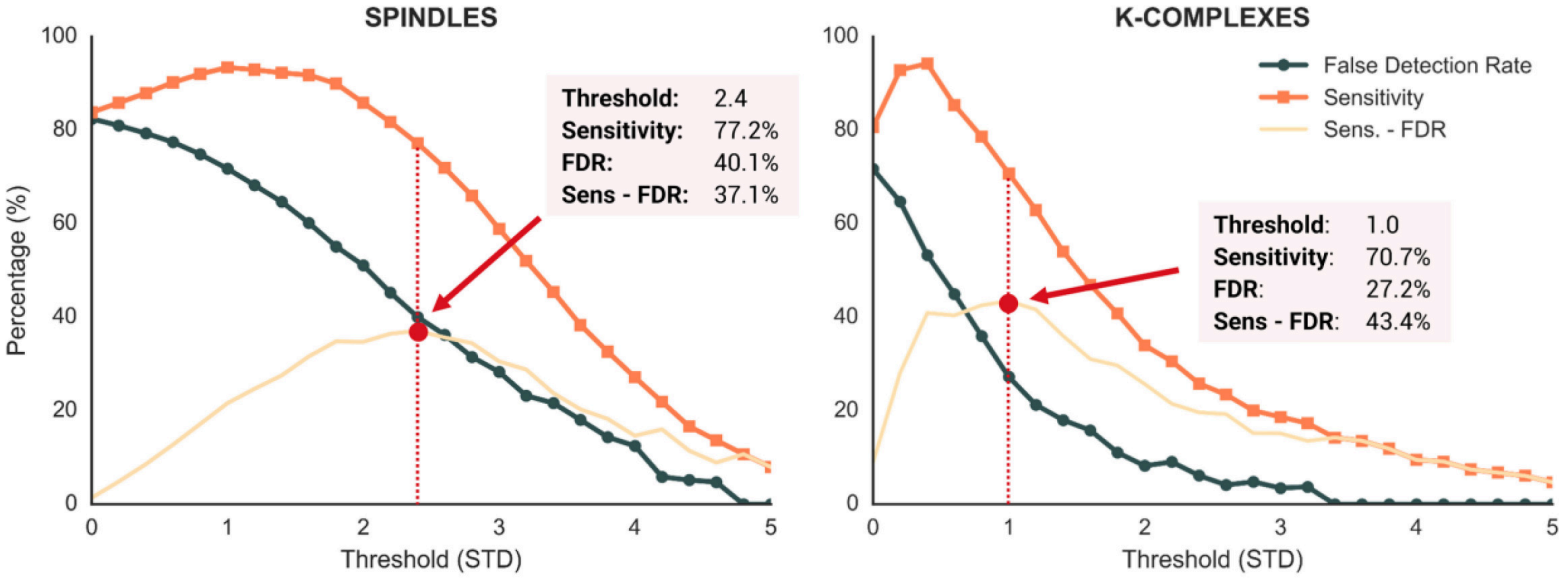
Performance metrics of the *Sleep* spindle and K-complex detection methods evaluated at 25 different thresholds (range = 0–5, step = 0.2). Dark orange and blue lines depict the sensitivity and false detection rate (FDR), respectively. Light orange lines show the difference between sensitivity and FDR. Red dotted lines depict the threshold values that maximized this difference.

Performances of the detection algorithm implemented in *Sleep* are reported in Figure 7. For both spindles and K-complexes, we used 25 different thresholds ranging from 0 to 5 with 0.2 steps. The optimal threshold was defined as the one that maximizes the difference between sensitivity and FDR (Lajnef et al., 2015a). Regarding spindles, the best performance of our algorithm was obtained at a threshold of 2.4 standard deviations, yielding a sensitivity of 77.2% and a FDR of 40.1%. Regarding K-complexes, a threshold of 1.0 resulted in the best performances with a sensitivity of 70.7% and a FDR of 27.2%. These results are similar to those of previous detection methods (Devuyst et al., 2011; Lajnef et al., 2015a). Moreover, the time of execution of these two algorithms are very fast.

### *Sleep* class inputs and code example

From a programming point of view, the high-level interface with our software is provided by the *Sleep* class. This class can take into account a few input arguments. Hence, loading sleep data can be assessed in three ways adapted to a range of users, from non-programmers to advanced users. As shown in the **Code Snippet 1**, running *Sleep* without further input arguments will ask the user to specify the path to a supported sleep dataset (^*^.eeg, ^*^.edf, or ^*^.trc). In addition, the user can either use an existing hypnogram or start a new one from scratch. Alternatively, instead of using the interface to select the files, they can be directly passed as input arguments (**Code Snippet 2**). In this example, we also demonstrate how to change the default order of the sleep stages in the hypnogram using a simple command-line option. If this option is not specified, the default display of *Sleep* is as follows: *Art, REM, Wake, N1, N2, N3*. Finally, several others file formats such as EEGLab, Neuroscan, EGI, GDF, and BDF can be loaded using MNE Python package^15^. We report in **Code Snippet 3** a method to pass data to *Sleep* after loading them using MNE python.

**Code Snippet 1** | Simplest way to launch Sleep from a Python interpreter. This will open a window asking the user to select the EEG data and corresponding hypnogram.


# Load the Sleep module from visbrain:
from visbrain import Sleep
# Open the default Sleep window:
Sleep().show()


**Code Snippet 2** | In this example, the paths to the EEG data and hypnogram are entered as inputs arguments of the main Sleep function, resulting in the software opening directly with the dataset and hypnogram loaded. We also show how to change the default display order of the hypnogram by changing the href argument of *Sleep* main function. The sleep stages will be displayed in the order defined in *norder* variable, with N3 on top and Art on bottom.


# Import the Sleep module from visbrain:
from visbrain import Sleep
# Define where the data are located:
dfile = '/home/perso/myfile.eeg'
# Define where the hypogram is located: hfile = '/home/perso/hypno.hyp'
# hfile = None # Eventually, start from a fresh one
# Inverse the default sleep stage order:
norder = ['n3', 'n2', 'n1', 'rem', 'wake', 'art']
# Finally, pass both file to the class:
Sleep(file=dfile, hypno_file=hfile, href=norder).show()


**Code Snippet 3** | This example shows a method to pass data to *Sleep* after loading them using MNE-Python package (see http://martinos.org/mne/dev/manual/io.html for a full list of the data formats supported by MNE)


# Import the Sleep module and MNE:
import numpy as np
from visbrain import Sleep
from mne import io, Annotations
# - Biosemi Data Format (BDF)
raw = io.read_raw_edf('mybdffile.bdf', preload=True)
# - EGI format
# raw = io.read_raw_egi('myegifile.egi', preload=True)
# - EEGLab
# raw = io.read_raw_eeglab('myeeglabfile.set', preload=True)
# Extract data, sampling frequency and channels names
data, sf, chan = raw._data, raw.info['sfreq'],
raw.info['ch_names']
# Define annotations for this file:
onset = np.array([145., 235., 1045.]) # Onset of each
event (sec)



dur = np.array([1., 5., 2.5]) # Duration (sec)

description = np.array(['First event', # Description

'Second event',

'Third event'

])

annot = Annotations(onset, dur, description)

# Now, pass all the arguments to the Sleep module:

Sleep(data=data, sf=sf, channels=chan, annotation_file=
annot).show()


## Discussion

This paper introduces an open-source software module called Sleep which provides a user-friendly and efficient GUI dedicated to visualization, scoring and analysis of sleep data. This proposed module is part of a larger ongoing open-source Python project by our group called *Visbrain* dedicated to the visualization of neuroscientific data. The design and functionalities of *Sleep* are specifically geared toward scientists and students involved in sleep research.

*Sleep* comes with a GUI in which we embedded high-quality plots with graphical rendering offloaded to the GPU. As a result, plotting and user interactions can be processed in real-time. The software is capable of loading several widely-used sleep data files format, such as European Data Format and BrainVision, and to stream efficiently all of the polysomnographic channels, even on an average modern laptop. On top of that, *Sleep* also provides the possibility to display time-frequency (spectrogram) and topographic representations of the data, with several adjustable parameters for each. Regarding sleep staging and hypnogram editing, *Sleep* offers intuitive manual scoring functionalities, signal processing tools and automatic detection of sleep features in order to facilitate this fastidious process. Once completed, users can export sleep statistics, or publication-ready high-quality figure of the hypnogram in one click.

### Comparison with other solutions

First, it is noteworthy that the scope and functionalities of the present module differs from a previous MATLAB tool we have released, called *Spinky* (Lajnef et al., 2017) and which aims specifically to provide a joint spindle and K-complex detection framework using the tunable Q-factor wavelet transform (TQWT) (Selesnick, 2011). In addition with being written entirely in Python, *Sleep* allows for a wide range of functionalities, such as sleep scoring, fast raw and spectral data visualization, edition and creation of hypnogram and annotation files, and automatic detection of several sleep features. *Spinky* and *Sleep* subserve distinct purposes and are thus highly complementary. Second, there are currently only a few freewares for human sleep scoring and analysis. The Python package Phypno and MATLAB-based toolbox SpiSOP both provide a GUI for scoring sleep stages, and include several other command line features to perform automatic detections and compute sleep statistics. However, one of the advantages of *Sleep* in comparison with these two solutions is the dynamic integration of these features into the GUI, which we believe will allow our software to be understood and accessible by users with no or little programming knowledge. Finally, *Sleep* offers several advantages compared to the numerous existing commercial solutions, the most obvious one being that it is free and therefore more easily accessible to students or small sleep laboratories. Also, the fact that it is open-source allows more easily the community to contribute to its extension and development. Furthermore, special emphasis was given to ensure compatibility with several electrophysiological and hypnogram file formats and thus liberate the data from proprietary formats that are dependent upon specific software. We firmly believe that this, in addition with the possibility to save and load automatic detection or configuration files, will promote and facilitate data sharing across sleep laboratories.

### Performance of the automatic detections

Regarding the automatic detections, *Sleep* includes 6 robust algorithms for detecting some of the most prominent features of each sleep stage, including spindles, K-complexes, slow waves, REM, and muscle twitches. Spindle and K-complex detection algorithms were validated on a visually scored dataset including 210 min of N2 sleep from 14 participants and resulted in performances similar to those reported in recent publications. Last but not least, these detections are implemented inside the GUI in an ergonomic and intuitive manner. We think that these detections may represent a valuable help not only in the process of staging sleep, but also for researchers that are interested in the microstructure of sleep. The automatic detection algorithms proposed in *Sleep* can be used as a starting point for a semi-automatic procedure where users can correct or adjust the output of the detector. Beyond saving a lot of time, this approach has generally been shown to yield reliable and robust detection (O'Reilly and Nielsen, 2015).

## Future directions and conclusion

We are considering to extend the list of the default supported files and we encourage programmers or sleep scientists interested by this project to collaborate on it. Regarding sleep analysis we are working on an automatic scoring function based on machine-learning algorithms, inline with our previous work (Combrisson and Jerbi, 2015; Lajnef et al., 2015b; Combrisson et al., 2017). Finally, as different users have different needs, we are constantly improving the interface and functionalities of the software thanks to the feedback we receive.

With the release of *Sleep*, we offer a portable and cross-platform software, installable and usable on most configuration. While there is still room for improvement, *Sleep* already provides a complete and intuitive interface designed by and for scientists involved in sleep research. We hope this software will be used and further developed by many like-minded students and researchers with a strong commitment to open science and to high quality open-source software.

## Author contributions

EC and RV contributed equally in the development of this software and writing of the article. JE provided visual scoring for the validation of K-complex and spindles detection. JE, CO, TL, AG, PR, and KJ actively helped in the writing process and with software testing.

### Conflict of interest statement

The authors declare that the research was conducted in the absence of any commercial or financial relationships that could be construed as a potential conflict of interest.

## Footnotes

^1^https://github.com/gpiantoni/phypno

^2^http://sleepsmg.sourceforge.net/

^3^http://spisop.org/

^4^http://vispy.org/

^5^https://github.com/EtienneCmb/visbrain

^6^https://riverbankcomputing.com/software/pyqt/intro

^7^https://www.wxwidgets.org/

^8^http://www.tcl.tk/

^9^https://www.qt.io/

^10^https://github.com/DiamondLightSource/auto_tomo_calibration-experimental/blob/master/old_code_scripts/peak_detect.py

^11^http://www.sphinx-doc.org/en/stable/

^12^http://visbrain.org/sleep.html

^13^http://visbrain.org/sleep.html#shortcuts

^14^http://visbrain.org/sleep.html#hypnogram

^15^https://martinos.org/mne/stable/manual/io.html#importing-eeg-data

## References

[B1] Berthomier C. Drouot X. Herman-Stoïca M. Berthomier P. Prado J. Bokar-Thire D.. (2007). Automatic analysis of single-channel sleep EEG: validation in healthy individuals. Sleep 30, 1587–1595. 10.1093/sleep/30.11.1587 18041491 PMC2082104

[B2] Billinger M. Brunner C. Müller-Putz G. R. (2014). SCoT: a Python Toolbox for EEG Source Connectivity. Available online at: https://pdfs.semanticscholar.org/b196/7f587fbea9ecf4cb6be3f757a8136fc60ca8.pdf 10.3389/fninf.2014.00022 PMC3949292 24653694

[B3] Campagnola L. Klein A. Larson E. Rossant C. Rougier N. P. (2015). VisPy: harnessing the GPU for fast, high-level visualization, in Proceedings of the 14th Python in Science Conference. Available online at: https://hal.inria.fr/hal-01208191/ (Accessed May 23, 2017).

[B4] Combrisson E. Jerbi K. (2015). Exceeding chance level by chance: the caveat of theoretical chance levels in brain signal classification and statistical assessment of decoding accuracy. J. Neurosci. Methods 250, 126–136. 10.1016/j.jneumeth.2015.01.010 25596422

[B5] Combrisson E. Perrone-Bertolotti M. Soto J. L. Alamian G. Kahane P. Lachaux J.-P.. (2017). From intentions to actions: neural oscillations encode motor processes through phase, amplitude and phase-amplitude coupling. Neuroimage 147, 473–487. 10.1016/j.neuroimage.2016.11.042 27915117

[B6] Devuyst S. Dutoit T. Stenuit P. Kerkhofs M. (2011). Automatic sleep spindles detection—overview and development of a standard proposal assessment method. Conf. Proc. IEEE Eng. Med. Biol. Soc. 2011, 1713–1716. 10.1109/IEMBS.2011.6090491 22254656

[B7] Eichenlaub J.-B. Bertrand O. Morlet D. Ruby P. (2014). Brain reactivity differentiates subjects with high and low dream recall frequencies during both sleep and wakefulness. Cereb. Cortex 24, 1206–1215. 10.1093/cercor/bhs388 23283685

[B8] Eichenlaub J.-B. Ruby P. Morlet D. (2012). What is the specificity of the response to the own first-name when presented as a novel in a passive oddball paradigm? An ERP study. Brain Res. 1447, 65–78. 10.1016/j.brainres.2012.01.072 22361115

[B9] Erdamar A. Duman F. Yetkin S. (2012). A wavelet and teager energy operator based method for automatic detection of K-complex in sleep EEG. Expert Syst. Appl. 39, 1284–1290. 10.1016/j.eswa.2011.07.138

[B10] Gramfort A. Luessi M. Larson E. Engemann D. A. Strohmeier D. Brodbeck C.. (2013). MEG and EEG data analysis with MNE-Python. Front. Neurosci. 7:267. 10.3389/fnins.2013.00267 24431986 PMC3872725

[B11] Iber C. Ancoli-Israel S. Chesson A. L. Quan S. F. (2007). The AASM Manual for the Scoring of Sleep and Associated Events: Rules, Terminology and Technical Specifications. Westchester, IL: American Academy of Sleep Medicine.

[B12] Jerbi K. Ossandón T. Hamamé C. M. Senova S. Dalal S. S. Jung J.. (2009). Task-related gamma-band dynamics from an intracerebral perspective: review and implications for surface EEG and MEG. Hum. Brain Mapp. 30, 1758–1771. 10.1002/hbm.20750 19343801 PMC6870589

[B13] Lajnef T. Chaibi S. Eichenlaub J.-B. Ruby P. M. Aguera P.-E. Samet M.. (2015a). Sleep spindle and K-complex detection using tunable Q-factor wavelet transform and morphological component analysis. Front. Hum. Neurosci. 9:414. 10.3389/fnhum.2015.00414 26283943 PMC4516876

[B14] Lajnef T. Chaibi S. Ruby P. Aguera P.-E. Eichenlaub J.-B. Samet M.. (2015b). Learning machines and sleeping brains: automatic sleep stage classification using decision-tree multi-class support vector machines. J. Neurosci. Methods 250, 94–105. 10.1016/j.jneumeth.2015.01.022 25629798

[B15] Lajnef T. O'Reilly C. Combrisson E. Chaibi S. Eichenlaub J.-B. Ruby P. M.. (2017). Meet spinky: an open-source spindle and K-complex detection toolbox validated on the open-access Montreal Archive of Sleep Studies (MASS). Front. Neuroinform. 11:15. 10.3389/fninf.2017.00015 28303099 PMC5332402

[B16] O'Reilly C. Nielsen T. (2015). Automatic sleep spindle detection: benchmarking with fine temporal resolution using open science tools. Front. Hum. Neurosci. 9:353. 10.3389/fnhum.2015.00353 26157375 PMC4478395

[B17] Parekh A. Selesnick I. W. Rapoport D. M. Ayappa I. (2015). Detection of K-complexes and sleep spindles (DETOKS) using sparse optimization. J. Neurosci. Methods 251, 37–46. 10.1016/j.jneumeth.2015.04.006 25956566

[B18] Rechtschaffen A. Kales A. (1968). A Manual of Standardized Terminology, Techniques and Scoring System for Sleep Stages of Human Subjects. Washington, DC: United States Government Printing Office.

[B19] Ruby P. Blochet C. Eichenlaub J.-B. Bertrand O. Morlet D. Bidet-Caulet A. (2013). Alpha reactivity to complex sounds differs during REM sleep and wakefulness. PLoS ONE 8:e79989. 10.1371/journal.pone.0079989 24260331 PMC3832371

[B20] Ruby P. M. Blochet C. Eichenlaub J.-B. Bertrand O. Morlet D. Bidet-Caulet A. (2013). Alpha reactivity to first names differs in subjects with high and low dream recall frequency. Front. Psychol. 4:419. 10.3389/fpsyg.2013.00419 23966960 PMC3743036

[B21] Selesnick I. W. (2011). Wavelet transform with tunable Q-factor. IEEE Trans. Signal Proc. 59, 3560–3575. 10.1109/TSP.2011.2143711

[B22] Tallon-Baudry C. Bertrand O. Delpuech C. Pernier J. (1996). Stimulus specificity of phase-locked and non-phase-locked 40 Hz visual responses in human. J. Neurosci. 16, 4240–4249. 8753885 10.1523/JNEUROSCI.16-13-04240.1996 PMC6579008

[B23] Vallat R. Lajnef T. Eichenlaub J.-B. Berthomier C. Jerbi K. Morlet D.. (2017). Increased evoked potentials to arousing auditory stimuli during sleep: implication for the understanding of dream recall. Front. Hum. Neurosci. 11:132. 10.3389/fnhum.2017.00132 28377708 PMC5360011

[B24] Warby S. C. Wendt S. L. Welinder P. Munk E. G. S. Carrillo O. Sorensen H. B. D.. (2014). Sleep-spindle detection: crowdsourcing and evaluating performance of experts, non-experts and automated methods. Nat. Methods 11, 385–392. 10.1038/nmeth.2855 24562424 PMC3972193

